# Dissecting the roles of peptidoglycan synthetic and autolytic activities in the walled to L-form bacterial transition

**DOI:** 10.3389/fmicb.2023.1204979

**Published:** 2023-06-02

**Authors:** Yoshikazu Kawai, Jeff Errington

**Affiliations:** Centre for Bacterial Cell Biology, Biosciences Institute, Medical School, Newcastle University, Newcastle upon Tyne, United Kingdom

**Keywords:** L-form bacteria, antibiotic resistance, *Bacillus subtilis*, cell wall, RodA, penicillin-binding proteins, LytE

## Abstract

Bacterial cells are surrounded by a peptidoglycan (PG) wall, which is a crucial target for antibiotics. It is well known that treatment with cell wall-active antibiotics occasionally converts bacteria to a non-walled “L-form” state that requires the loss of cell wall integrity. L-forms may have an important role in antibiotic resistance and recurrent infection. Recent work has revealed that inhibition of *de novo* PG precursor synthesis efficiently induces the L-form conversion in a wide range of bacteria, but the molecular mechanisms remain poorly understood. Growth of walled bacteria requires the orderly expansion of the PG layer, which involves the concerted action not just of synthases but also degradative enzymes called autolysins. Most rod-shaped bacteria have two complementary systems for PG insertion, the Rod and aPBP systems. *Bacillus subtilis* has two major autolysins, called LytE and CwlO, which are thought to have partially redundant functions. We have dissected the functions of autolysins, relative to the Rod and aPBP systems, during the switch to L-form state. Our results suggest that when *de novo* PG precursor synthesis is inhibited, residual PG synthesis occurs specifically *via* the aPBP pathway, and that this is required for continued autolytic activity by LytE/CwlO, resulting in cell bulging and efficient L-form emergence. The failure of L-form generation in cells lacking aPBPs was rescued by enhancing the Rod system and in this case, emergence specifically required LytE but was not associated with cell bulging. Our results suggest that two distinct pathways of L-form emergence exist depending on whether PG synthesis is being supported by the aPBP or RodA PG synthases. This work provides new insights into mechanisms of L-form generation, and specialisation in the roles of essential autolysins in relation to the recently recognised dual PG synthetic systems of bacteria.

## Introduction

The peptidoglycan (PG) cell wall is an essential structure for most bacteria and the target for many of our most effective antibiotics. The wall is composed of glycan strands cross-linked by short peptides, forming a huge contiguous meshwork that covers the whole surface of the cell. Expansion of the cell during growth requires the insertion of new PG, which is achieved by the action of glycosyltransferase (GTase) enzymes, to extend the glycan chains, and transpeptidase (TPase) enzymes, which introduce cross-links (Typas et al., 2012; Egan et al., 2020). Recent work has established that most bacteria have two complementary PG insertion systems (Meeske et al., 2016; Emami et al., 2017; Sjodt et al., 2018; Dion et al., 2019; Garner, 2021). The Rod system involves GTases called RodA and FtsW, which work in conjunction with cognate monofunctional TPases called class B penicillin-binding proteins (bPBPs) (Reichmann et al., 2019; Taguchi et al., 2019; Sjodt et al., 2020). They are regulated spatially and temporally to achieve orderly cell extension and then division, governed by cytoskeletal proteins MreB and FtsZ, respectively (Typas et al., 2012; Egan et al., 2020). The aPBP system is based on bifunctional class A PBPs (aPBPs) that have both GTase and TPase activity. In *Bacillus subtilis* the aPBP system seems to insert new PG in a dispersed manner leading, in the absence of the Rod system, to growth in a spherical form. However, the precise function of the aPBP system is not clear, and all four aPBPs (*ponA*, *pbpD*, *pbpF* and *pbpG*) can actually be deleted with only mild effects on cell growth and morphology (McPherson and Popham, 2003; Emami et al., 2017; Dion et al., 2019).

Cell wall growth invariably relies also on the action of PG hydrolases, sometimes called autolysins, which break bonds in the PG meshwork. These enzymes are extremely abundant and redundant, *B. subtilis* has 42 putative autolytic enzymes (Wilson and Garner, 2021). However, their specific functions are poorly characterised. Mutations in several of the genes have mild effects on cell separation after division, and mutations in multiple genes tend to have additive effects, suggesting that they have overlapping functions (Fukushima et al., 2006; Wilson and Garner, 2021). For cell elongation, it has been established that two genes *lytE* and *cwlO*, encoding D,L-endopeptidase autolysins, have important and overlapping functions: single deletions have no significant effect on cell growth but the double mutant is inviable, with an apparent complete arrest in cell elongation (Bisicchia et al., 2007; Hashimoto et al., 2012; Meisner et al., 2013; Salzberg et al., 2013; Wilson and Garner, 2021). It has long been assumed that the main role of autolytic enzymes during cell elongation is to break bonds in PG, so as to enable the insertion of new wall material. However, their activities also clearly need to be tightly regulated because autolysins can also be the source of catastrophic cell lysis (Höltje, 1998). Recent studies have started to explore the regulation of autolysins, and the relationships between autolysins and the Rod and aPBP synthetic systems (Dobihal et al., 2019; Patel et al., 2020), although much remains to be understood.

Despite the critical importance of the PG wall for the cell viability, under osmoprotective conditions many bacteria have the ability to switch into a wall-deficient state called the L-form (Klieneberger, 1935), which is completely resistant to antibiotics that work on cell-wall-active antibiotics, such as β-lactams. Historically L-forms have mainly been identified as antibiotic resistant organisms in samples from humans, and have been associated with a wide range of infectious diseases (Domingue and Woody, 1997; Allan et al., 2009; Errington et al., 2016). Our recent findings have provided mechanistic support for the view that L-forms may be involved in chronic or recurrent infections (Kawai et al., 2018; Mickiewicz et al., 2019). However, despite their critical importance for understanding antibiotic resistance and pathogenesis, molecular mechanisms underlying the generation of L-forms are still poorly understood.

We have previously shown that, provided *B. subtilis* cells have a mutation that downgrades the respiratory chain pathway such as *ispA* (which works to avoid generation of toxic reactive oxygen species) and are on an isotonic medium, they can avoid killing by antibiotics that inhibit synthesis of the PG precursor, lipid II, by switching into the wall-deficient L-form state (Mercier et al., 2013; Kawai et al., 2015, 2019). The antibiotic D-cycloserine (DCS), which inhibits D-alanine-D-alanine ligase in the lipid II pathway, efficiently induces such a switch. L-form production induced by DCS occurs *via* a gradual bulging of the walled cell before the L-form emerges (Kawai et al., 2018). We call this critical event in the formation of L-forms “escape.” Escape is dependent on the aPBP PG synthases, as well as the autolytic enzymes, LytE and CwlO (Kawai et al., 2018). Thus, deletion of all four aPBPs (encoded by *ponA*, *pbpD*, *pbpF* and *pbpG* genes) or the combined absence of LytE and CwlO inhibits L-form escape (Kawai et al., 2018). In contrast, escape is not blocked by mutations affecting RodA. The L-form escape system therefore provides a novel platform for studying the connections between PG synthetic and hydrolytic activities and between the RodA and aPBP systems. Here, we have exploited this system to obtain important new insights into these connections. The results shed light on specialisation of the functions of CwlO and LytE and of the RodA vs. aPBP systems during L-form escape in *B. subtilis*. They also enhance our understanding of factors that can affect the ability of cells to undertake the L-form switch that results in the evasion of killing by important antibiotics.

## Materials and methods

### Strains and growth conditions

Nutrient agar and broth (Oxoid) were used for *B. subtilis* growth. All strains used in this work are listed in Table 1. DNA manipulations and transformations were carried out using standard methods. For selections of transformants, cells were plated on NA plates supplemented with 1 μg/mL erythromycin, 5 μg/mL chlroramphenicol, 60 μg/mL spectinomycin, 1 μg/mL phleomycin, 2.5 μg/mL kanamycin and/or 10 μg/mL tetracycline. IPTG, xylose or Mg^2+^ was supplemented, as appropriate. *ΔmreB* and *Δmbl* mutants were selected on NA containing added 10 mM MgSO_4_. *ΔrodA* mutant was selected on isotonic NA plates, composed of 2x magnesium-sucrose-maleic acid (MSM) pH7 (40 mM magnesium chloride, 1 M sucrose, and 40 mM maleic acid) mixed 1:1 with 2x NA. The concentration of kanamycin or tetracycline was increased to 10 μg/mL or 30 μg/mL in the presence of added Mg^2+^. Since transformation in *Δ4* mutant cells was less effective, an temperature sensitive unstable pLOSS-*P_spac_*-*ponA* plasmid was used to improve the transformation efficiency by providing *ponA* expression with IPTG in *Δ4* mutant backgrounds (Claessen et al., 2008; Emami et al., 2017). Antibiotics were added to media for growth of strains carrying Ω*erm*-*∆cwlO*, Ω*erm-P_spac_*-*ispA*, pLOSS-*P_spac_*-*ponA*, Ω*kan-P_spac_*-*rodA* or Ω*kan*-*P_spac_*-*ftsW* at the following concentrations: 1 μg/mL erythromycin, 60 μg/mL spectinomycin or 2.5 or 10 μg/mL kanamycin.

**Table 1:** *Bacillus subtilis* strains.

Strains	Genotypes	References
168CA	*trpC2*	Lab stock
AG221	*trpC2 ∆ponA ∆pbpD ∆pbpF ∆pbpG*::*erm* pLOSS-*P_spac_*-*ponA*-*spc*	Emami et al. (2017)
LR2	*trpC2* Ω*murE*::*cat*-*P_xyl_*-*murE ispA^−^*	Mercier et al. (2013)
YK1395	trpC2 ispA^*^ (*xseB*::*Tn*-*kan*)	Mercier et al. (2013)
YK1559	*trpC2* Ω*glmM*::*erm*-*P_spac_*-*glmM*	Kawai et al. (2019)
YK1859	trpC2 ispA^*^ (*xseB*::*Tn*-*kan*) *P_spac_-ftsZ*-*phleo*	Leaver et al. (2009) and Mercier et al. (2013)
YK1874	trpC2 ispA^*^ (*xseB*::*Tn*-*kan*) *∆mreB*::*spc ∆mbl ∆mreBH amyE*::*_spacHY_*-*mbl*-*cat*	Kawai et al. (2009, 2011) and Mercier et al. (2013)
YK1876	trpC2 ispA^*^ (*xseB*::*Tn*-*kan*) Ω*neo-∆mreB*-*mreC*-*mreD amyE*::*P_xyl_*-*mreB*-*mreC*-*mreD*-*spc*	Leaver and Errington (2005) and Mercier et al. (2013)
YK2239	*trpC2 ΔponA ΔpbpD ΔpbpF ΔpbpG*::*erm*	Emami et al. (2017)
YK2283	*trpC2 ΔponA ΔpbpD ΔpbpF ΔpbpG*::*erm amyE::P_xyl_-rodA*-*cat*	Emami et al. (2017)
YK2342	*trpC2 ΔponA ΔpbpD ΔpbpF ΔpbpG*::*erm* Ω*murE*::*cat*-*P_xyl_*-murE ispA^*^ (*xseB*::*Tn*-*kan*)	Mercier et al. (2013) and Emami et al. (2017)
YK2344	trpC2 ispA^*^ (*xseB*::*Tn*-*kan*) *∆ponA ∆pbpD ∆pbpF ∆pbpG*::*cat*	Mercier et al. (2013) and Emami et al. (2017)
YK2483	*trpC2 ∆rodA*::*kan* Ω*ispA*::*erm*-*P_spac_*-*ispA*	Kawai et al. (2011, 2015)
YK2515	*trpC2 ΔponA ΔpbpD ΔpbpF ΔpbpG*::*kan amyE::P_xyl_-rodA*-*cat* Ω*ispA*::*erm*-*P_spac_*-*ispA*	Kawai et al. (2015) and Emami et al. (2017)
YK2524	trpC2 ispA^*^ (*xseB*::*Tn*-*kan*) *∆lytE*::*tet* Ω*cwlO*::*erm*-*P_spac_*-*cwlO*	Mercier et al. (2013) and Kawai et al. (2018)
YK2526	*trpC2 ∆lytE*::*tet ΔponA ΔpbpD ΔpbpF ΔpbpG*::*kan amyE::P_xyl_-rodA*-*cat* Ω*ispA*::*erm*-*P_spac_*-*ispA*	Domínguez-Cuevas et al. (2013), Kawai et al. (2015) and Emami et al. (2017)
YK2527	*trpC2 ∆cwlO*::*spc ΔponA ΔpbpD ΔpbpF ΔpbpG*::*kan amyE::P_xyl_-rodA*-*cat* Ω*ispA*::*erm*-*P_spac_*-*ispA*	Domínguez-Cuevas et al. (2013), Kawai et al. (2015) and Emami et al. (2017)
YK2535	*trpC2 Δmbl*::*spc ΔponA ΔpbpD ΔpbpF ΔpbpG*::*kan amyE::P_xyl_-rodA*-*cat* Ω*ispA*::*erm*-*P_spac_*-*ispA*	Schirner and Errington (2009), Kawai et al. (2015) and Emami et al. (2017)
YK2538	*trpC2 ΔmreBH*::*spc ΔponA ΔpbpD ΔpbpF ΔpbpG*::*kan amyE::P_xyl_-rodA*-*cat* Ω*ispA*::*erm*-*P_spac_*-*ispA*	Schirner and Errington (2009), Kawai et al., 2015 and Emami et al. (2017)
YK2574	trpC2 ispA^*^ (*xseB*::*Tn*-*kan*) *P_spac_-ftsW*-*kan*	Mercier et al. (2013) and Gamba et al. (2016)
YK2586	*trpC2 ΔponA ΔpbpD ΔpbpF ΔpbpG*::*kan* Ω*glmM*::*erm*-*P_spac_*-*glmM*	Kawai et al. (2015) and Emami et al. (2017)
YK2560	*trpC2 ∆lytE*::*tet ∆ponA ∆pbpD ∆pbpF ∆pbpG*::*kan* pLOSS-*P_spac_*-*ponA*-*spc*	Domínguez-Cuevas et al. (2013) and Emami et al. (2017)
YK2562	*trpC2* Ω*erm*-*∆cwlO ∆ponA ∆pbpD ∆pbpF ∆pbpG*::*kan* pLOSS-*P_spac_*-*ponA*-*spc*	Emami et al. (2017)
YK2564	*trpC2 ∆lytE*::*tet ∆ponA ∆pbpD ∆pbpF ∆pbpG*::*kan*	Domínguez-Cuevas et al. (2013) and Emami et al. (2017)
YK2596	*trpC2 ΔmreB ΔponA ΔpbpD ΔpbpF ΔpbpG*::*kan amyE::P_xyl_-rodA*-*cat* Ω*ispA*::*erm*-*P_spac_*-*ispA*	Kawai et al. (2011, 2015) and Emami et al. (2017)

Resistance gene abbreviations are as follows; erm, erythromycin; spc, spectinomycin; kan, kanamycin; phleo, pheleomycin; cat, chloramphenicol; neo, neomycin; tet, tetracycline.

### L-form escape and growth

L-form growth was induced on osmoprotective plates (NA containing MSM) in the presence of 200 μg/mL D-cycloserine (DCS) or genetic inhibition of PG precursor synthesis at 30°C. 1 μg/mL FtsZ inhibitor, 8 J (Adams et al., 2011), was used in L-form culture to prevent the growth of walled cells when required. IPTG or xylose was supplemented, as appropriate. For L-form escape experiments in liquid media, walled cells were pre-cultured in NB at 37°C. The exponentially growing cells (OD_600_ 0.2–0.3) were diluted in the fresh osmoprotective medium (NB containing MSM) with 200 μg/mL D-cycloserine (DCS) or genetic inhibition of PG precursor synthesis, and incubated without shaking at 30°C.

### Microscopic imaging

For snapshot live cell imaging, walled cells were mounted on microscope slides covered with a thin film of 1.2% agarose in water, essentially as described previously (Glaser et al., 1997). L-form cultures were mounted on microscope slides covered with MSM. All microscopy experiments were conducted using a Nikon Ti microsope equipped with a Nikon CFI Plan Apo DM Lambda x100 oil objective and a Photometrics Prime camera, using MetaMorph software (version 7.7, Molecular Devices). Images were analysed and processed using FIJI.^1^

## Results

### L-form escape Is independent of various components of The rod system

**Figure 1:**
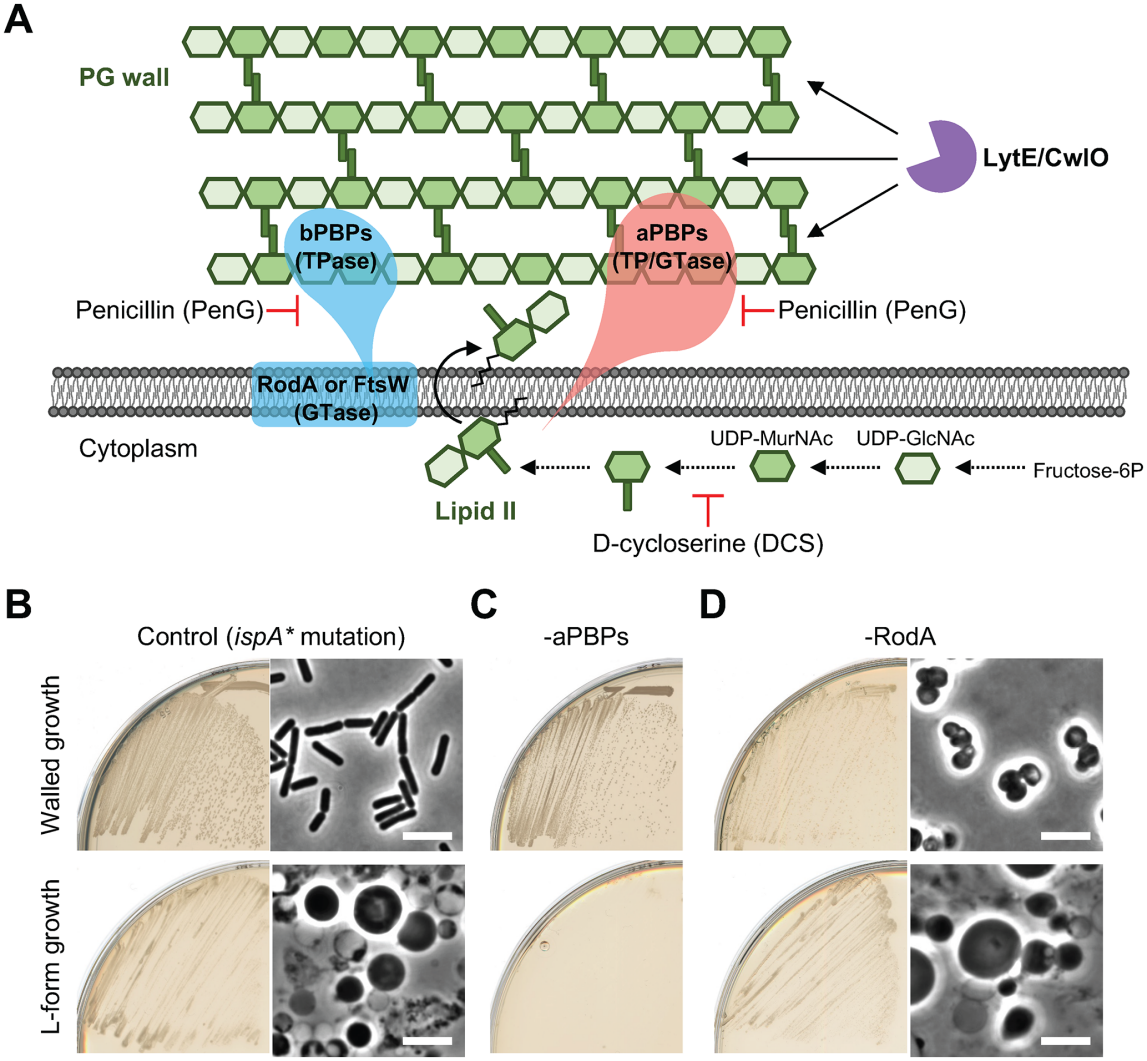
L-form generation requires aPBPs but not RodA **(A)** Schematic representation of cell wall expansion by dual PG synthetic systems and major autolysins during growth of *Bacillus subtilis*. PG precursor, lipid II, can be incorporated into the existing PG wall by the Rod and/or aPBP PG synthetic systems. The action of PG hydrolases (LytE and CwlO) is also essential for the PG growth during cell elongation. **(B-D)** Walled cell and L-form growth of strains YK1395 (ispA*), YK2344 (Δ4 ispA*) and YK2483 (*ΔrodA P_spac_-ispA*). The strains were streaked on isotonic NA plates with or without 200 μg/mL DCS and incubated at 30°C for 1 day (walled growth without DCS) or 2–3 days (L-form growth with DCS). Phase contrast micrographs were taken from colonies on the plates. Scale bars represent 5 μm.

We previously found that L-form escape following the inhibition of de novo PG precursor synthesis requires aPBPs, but not RodA (Figure 1A; Kawai et al., 2018). Figure 1B shows growth and morphology of otherwise wild type *B. subtilis* in the presence of an ispA* mutation (*xseB::Tn-kan*) that prevents IspA synthesis (Mercier et al., 2013). We used *ispA* mutations in all of the L-form experiments in this work so as to enable stable L-form growth by reducing the generation of toxic ROS (Kawai et al., 2015). The cells were cultured on isotonic nutrient agar (NA) plates containing osmoprotective sucrose and extra Mg^2+^ with (lower panels) or without (upper panels) D-cycloserine (DCS), which inhibits the PG precursor pathway (Figure 1A). Under these conditions, deletion of all four aPBPs (“*Δ4* mutant”) prevented the switch to the L-form state (Figure 1C). In contrast, L-form growth occurred significantly in the absence of RodA (Figure 1D, lower panels), whereas it resulted in a spherical mode of growth in the walled state (upper panels) (RodA is normally essential in the walled state but growth can be rescued by the addition of osmolytes) (e.g., sucrose and Mg^2+^ to standard culture media) (Kawai et al., 2011).

**Figure 2:**
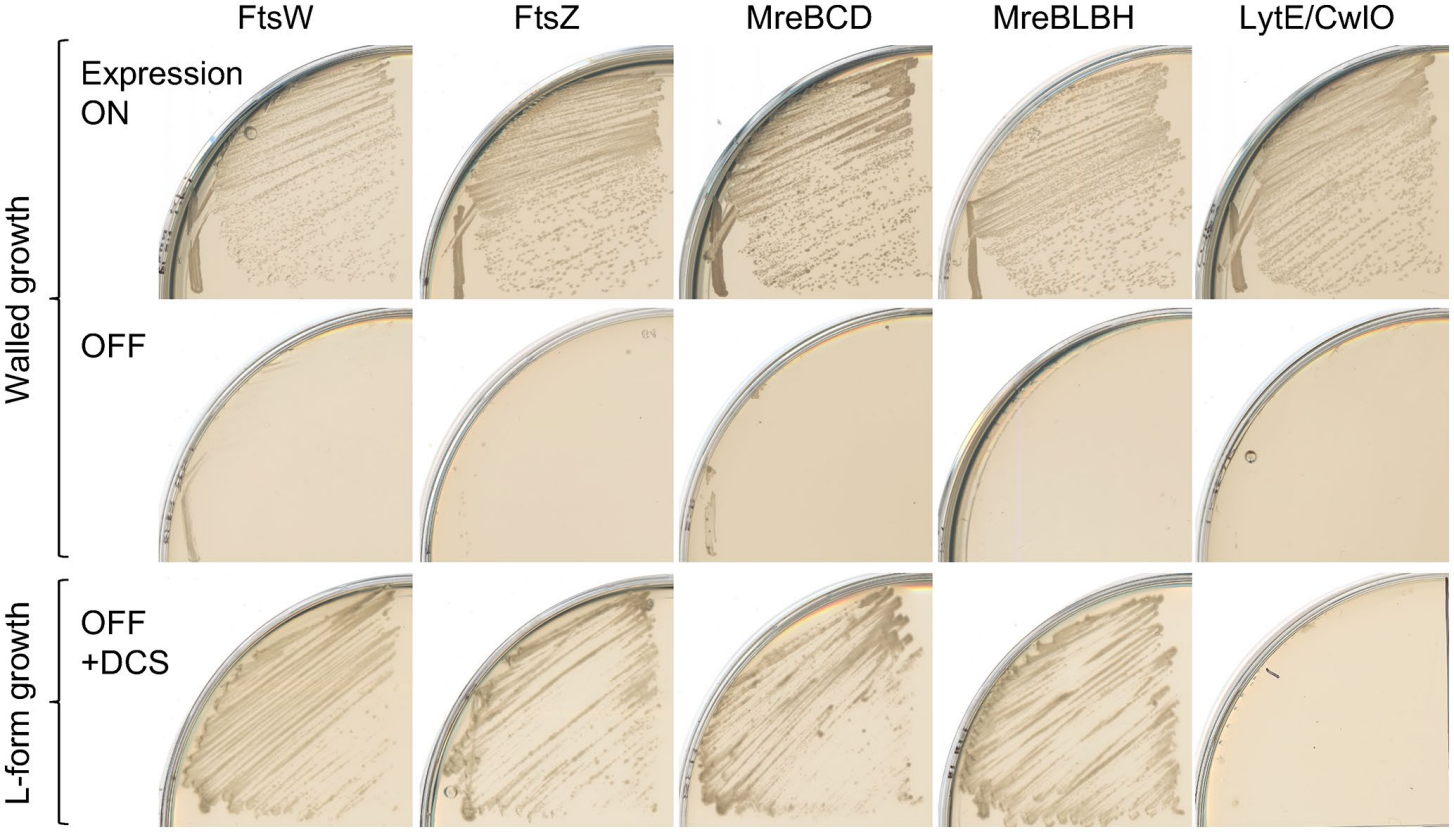
L-form generation does not require the Rod system Walled cell and L-form growth of strains YK2574 (P_spac_-ftsW ispA^*^; FtsW), YK1859 (P_spac_-ftsZ ispA^*^; FtsZ), YK1876 (*ΔmreB ΔmreC ΔmreD* *amyE*::*P_xyl_-mreB*-*mreC*-*mreD* *ΔispA*; MreBCD) and YK1874 (ΔmreB Δmbl ΔmreBH amyE::P_spacHY_-mbl ispA^*^; MreBLBH) and YK2524 (ΔlytE P_spac_-cwlO ispA^*^; LytE/CwlO) on normal NA plates with or without 0.5 mM IPTG (or 0.5% xylose for YK1876) at 30°C for 1  day (Walled growth), or on isotonic NA plates (containing added sucrose and Mg^2+^) with 200  μg/mL DCS at 30°C for 2–3  days (L-form growth).

We tested for the effects of repression of other morphogenic proteins required for cell elongation or division (brought about by repression of these genes under IPTG or xylose control). Repression of the division specific PG synthase FtsW did not affect escape (Figure 2, FtsW lower left). L-form escape also occurred efficiently in the absence of the division complex organiser FtsZ, or of the elongation organiser MreB, whether it was eliminated along with its “partners” (MreC and MreD) or its isoforms (Mbl and MreBH) (Figure 2, lower panels). In contrast, the combined absence of LytE and CwlO inhibited L-form growth (Figure 2, lower right), as previously shown (Kawai et al., 2018). All of these mutations abolished growth under selection for the walled state (without added sucrose and Mg^2+^) (Figure 2, OFF, middle panels), as expected. Thus, L-form escape seems to depend specifically on the aPBP pathway and the autolytic activity of either LytE or CwlO.

### Dispersed PG synthesis by the aPBP pathway during PG precursor depletion

**Figure 3:**
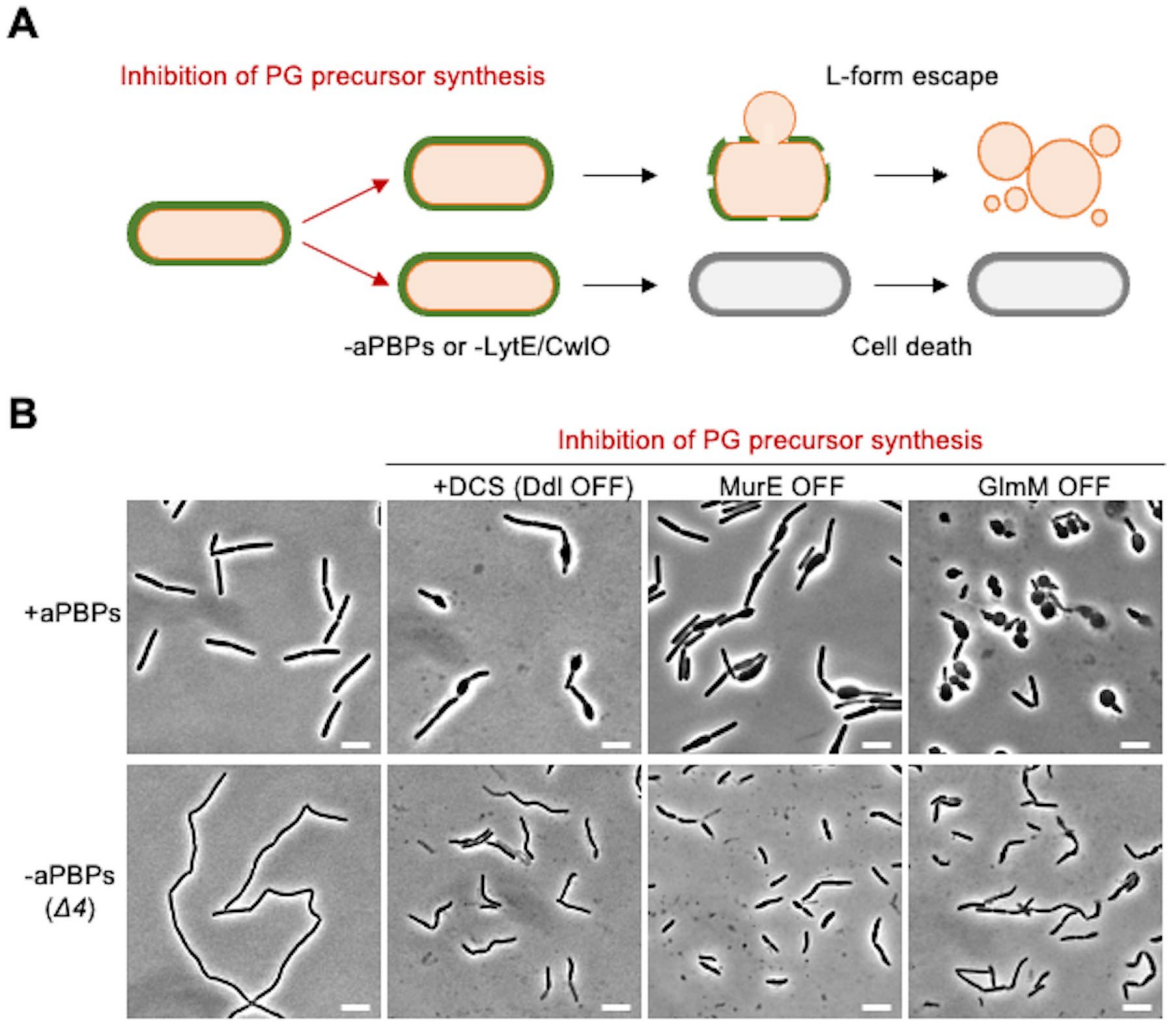
Crucial role for the aPBP pathway in bulging when PG precursors are limiting **(A)** Schematic representation of L-form escape during PG precursor depletion in *B. subtilis*. Emergence of L-form cells induced by inhibition of PG precursor synthesis occurs in association with bulging of walled cells. Inactivation of all four aPBPs (*Δ4*) or the combined absence of LytE and CwlO inhibits the bulging formation and L-form emergence. **(B)** Phase contrast micrographs of strains YK1395 (ispA*), YK2344 (Δ4 ispA*), LR2 (P_xyl_-murE ispA*), YK2342 (Δ4 P_xyl_-murE ispA*), YK1559 (*P_spac_-glmM*) and YK2586 (*Δ4 P_spac_-glmM*). The strains YK1395 and YK2344 were cultured in isotonic liquid nutrient broth (NB) at 37°C. The exponentially growing cells were diluted in the fresh isotonic NB with or without 200 μg/mL DCS, and the phase contrast micrographs were captured by 2–3 h after the dilutions. The strains LR2, YK2342, YK1559 and YK2586 were cultured in isotonic NB with or without 0.2% xylose (for *murE* expression) or 0.1 mM IPTG (for *glmM* expression) at 37°C. The exponentially growing cells were diluted in the fresh isotonic NB (without xylose and IPTG) and the phase contrast micrographs were captured by 2–3 h after the dilutions (MurE OFF and GlmM OFF). Scale bars represent 5 μm.

We have previously shown that L-form escape induced by DCS occurs *via* a gradual bulging of the walled cell before the L-form emerges (Figure 3A; Kawai et al., 2018). Our previous work has also shown that the aPBP system and the autolytic activity of either LytE or CwlO are needed for the bulging and L-form escape in the presence of DCS (Figure 3B). The bulging can also be induced by repressing genes acting in the PG precursor pathway, for example, the *murE* operon (Figure 3B, MurE OFF) (Kawai et al., 2018). GlmM protein catalyses a key step in the synthesis of uridine 5′-diphospho-N-acetylglucosamine (UDP-GlcNAc), which is an essential intermediate in the lipid II pathway (Figure 1A). The repression of GlmM again induced bulging (Figure 3B, GlmM OFF). Crucially, under each of these conditions, bulging of the walled cells was blocked by deletion of the aPBP genes (Figure 3B, lower panels). Thus, it seems that under conditions of limiting PG precursors, the aPBP pathway takes precedence over the Rod pathway, leading to dispersed PG synthesis and bulging.

### RodA overproduction compensates for the loss of aPBPs in L-form generation

**Figure 4:**
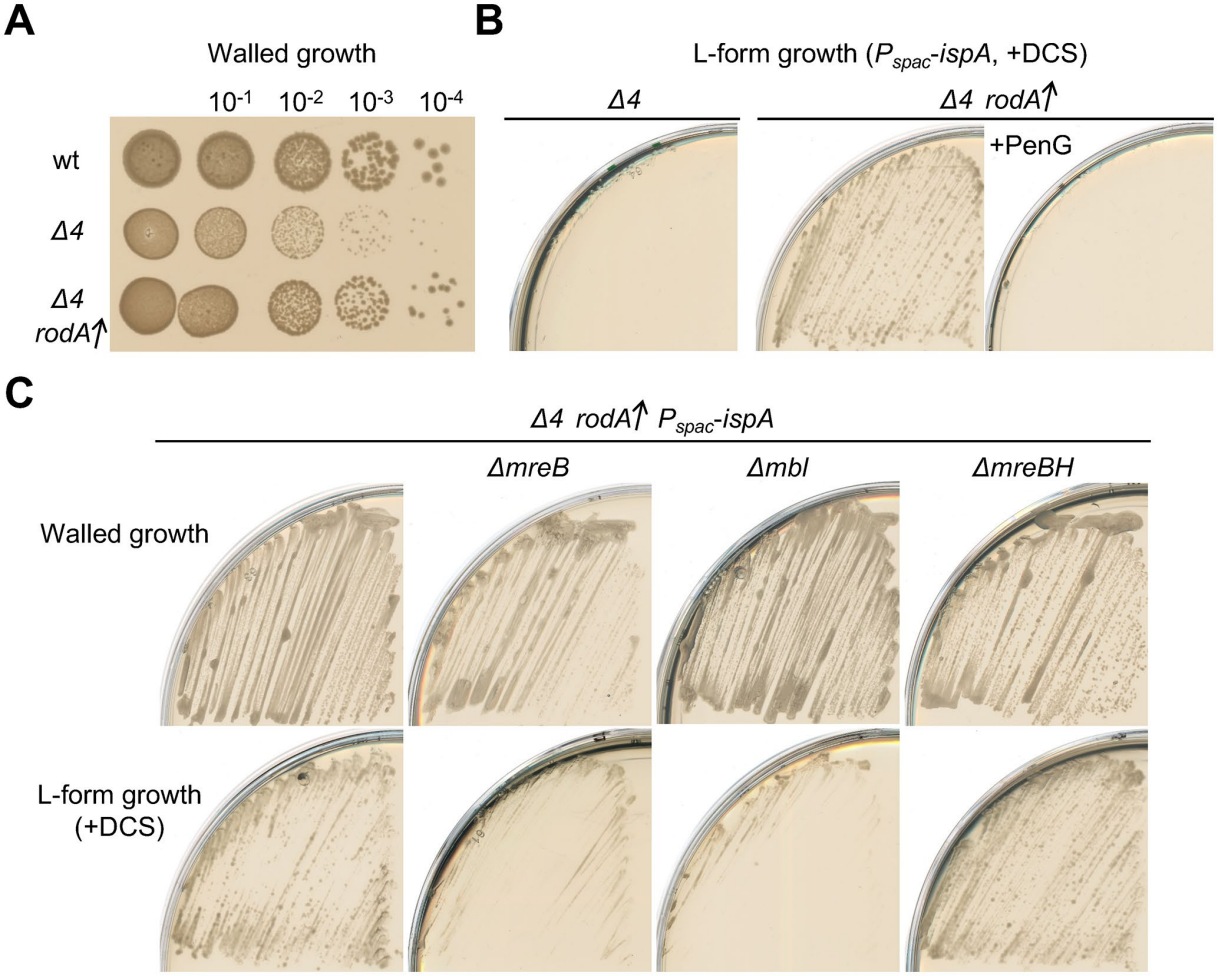
Induction of L-form generation by the Rod pathway **(A)** Exponentially growing cells of strains 168CA (wild-type), YK2239 (*Δ4*) and YK2283 (*Δ4 amyE*::*P_xyl_*-*rodA*) in NB were diluted (tenfold series) and 6 μL spots were placed on NA plates with 0.5% xylose, and incubated at 30°C for 1 day. **(B)** L-form growth of strain YK2515 (*Δ4 amyE*::*P_xyl_*-*rodA P_spac_*-*ispA*). The strain YK2515 was streaked on isotonic NA plates with or without 1% xylose (for *rodA* overexpression) in the presence of 200 μg/mL DCS, and incubated at 30°C for 2–3 days. 100 μg/mL PenG was also added to the medium as indicated (right panel). **(C)** Walled cell and L-form growth of strains YK2515 (*Δ4 amyE*::*P_xyl_*-*rodA P_spac_*-*ispA*), YK2596 (*ΔmreB Δ4 amyE*::*P_xyl_*-*rodA P_spac_*-*ispA*), YK2535 (*Δmbl Δ4 amyE*::*P_xyl_*-*rodA P_spac_*-*ispA*) and YK2538 (*ΔmreBH Δ4 amyE*::*P_xyl_*-*rodA P_spac_*-*ispA*). The strains were streaked on isotonic NA plates containing 1% xylose with or without 200 μg/mL DCS, and incubated at 30°C for 2–3 days (L-form growth with DCS) or 1 day (walled cell growth without DCS).

Previous reports showed that upregulation of *rodA* can ameliorate growth deficiencies in walled cells caused by the inactivation of aPBPs under certain conditions (e.g., moenomycin treatment, which blocks aPBP GTase activity) (Meeske et al., 2016; Emami et al., 2017). Consistent with this, overproduction of RodA, using a second copy of the *rodA* gene under xylose control at an ectopic locus (*amyE*::*P_xyl_*-*rodA*), improved the slow growth phenotype that is typical of *Δ4* (aPBP) mutants (Figure 4A). We wondered if *rodA* upregulation might also rescue the defect in L-form escape of a *Δ4* mutant. To test this we built a derivative of the *Δ4* (*ispA* repressible) strain containing the extra copy of *rodA* (*P_xyl_-rodA*). We grew this strain with and without xylose and then streaked the cells on L-form selective plates containing DCS. Cells grown with overproduced RodA gave rise to an abundance of L-form colonies (Figure 4B, middle), whereas no growth occurred for the uninduced cells (Figure 4B, left). Thus, RodA overproduction works not only to compensate for the loss of aPBPs in growth of walled cells but also for L-form escape.

### L-form generation by the rod pathway in the absence of aPBPs

PG synthesis by the RodA GTase depends on the TPase activity of its cognate bPBPs, PbpA and/or PbpH (Wei et al., 2003; Dion et al., 2019; Sjodt et al., 2020). The TPase activity of bPBPs, like that of aPBPs, is targeted by β-lactam antibiotics (Figure 1A; Lovering et al., 2012). If the rescue of L-form growth in the absence of aPBPs by RodA overproduction worked by compensating for the loss of PG synthesis, the effect should be blocked by the broad targeting β-lactam penicillin G. This was indeed the case (Figure 4B, right). Thus, escape by this pathway requires not only increased levels of the RodA GTase but also at least one of the cognate bPBPs, and probably other components of the Rod elongation system.

We introduced mutations of the elongation organiser MreB and its isoforms (*ΔmreB*, *Δmbl* and *ΔmreBH*) separately into a *Δ4* strain carrying an ectopic copy of P_xyl_-*rodA*. No strong effect of those deletions on normal walled growth was observed on isotonic L-form plates, that contain excess Mg^2+^ (Figure 4C, upper panels) (The growth deficiency of *mreB* and *mbl* mutants under standard conditions [e.g. NA] can be rescued by the addition of high concentrations of Mg^2+^ (Formstone and Errington, 2005; Leaver and Errington, 2005; Schirner and Errington, 2009) In the presence of DCS, although no significant effect on L-form growth was seen in the *ΔmreBH* strain, deletion of either of the “major” MreB encoding genes, *mreB* or *mbl*, impaired the emergence of L-form colonies (Figure 4C, lower panels), supporting the idea that the escape in a *Δ4* RodA overproducing background requires function of the Rod elongation complex.

### A distinct form of L-form escape by the Rod pathway

**Figure 5:**
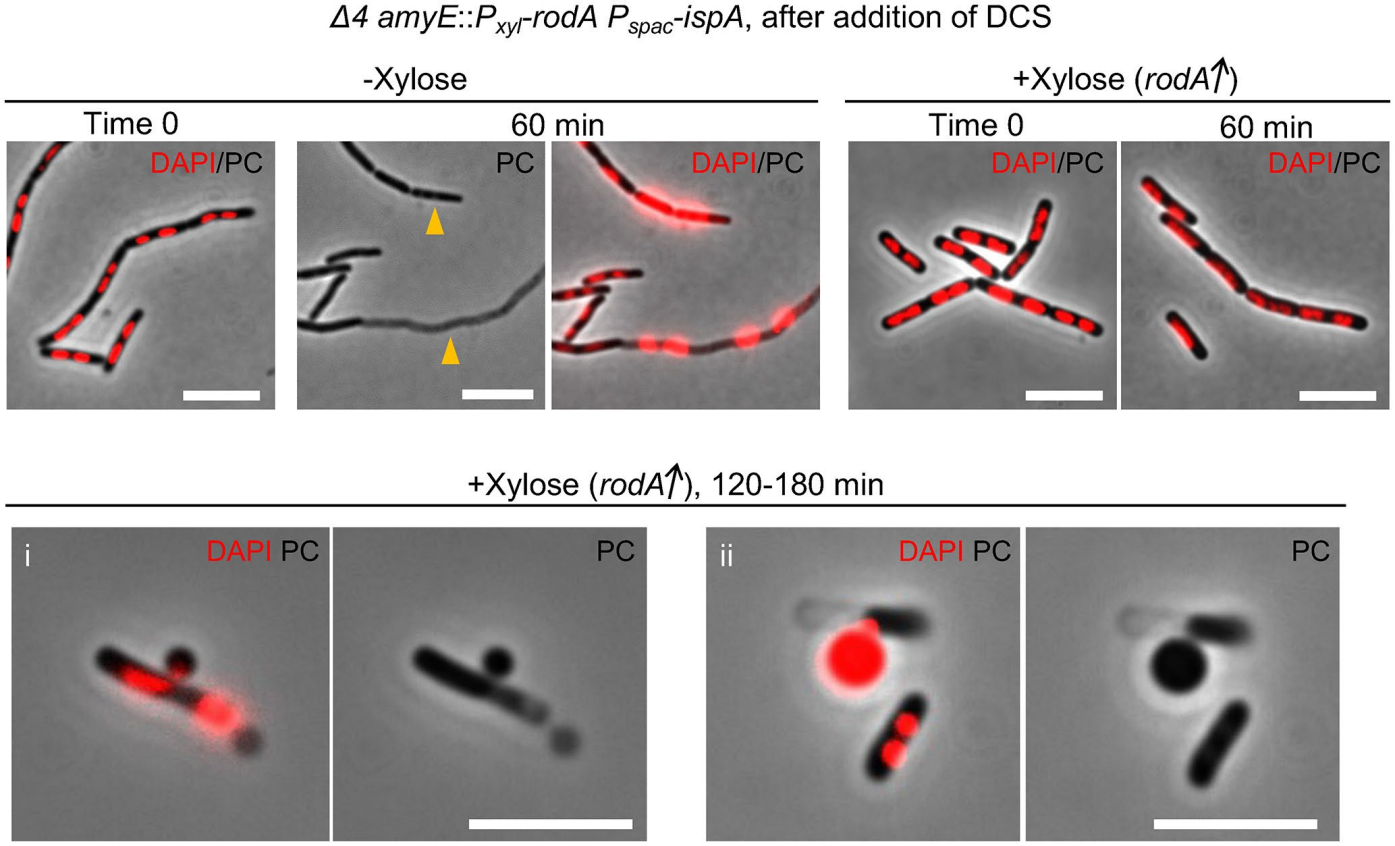
L-form escape by the Rod pathway Time course observation of nucleoid dynamics and cell morphology of strain YK2515 (*Δ4 amyE*::*P_xyl_*-*rodA P_spac_*-*ispA*). The YK2515 was cultured in isotonic NB with or without 0.5% xylose at 37°C and the exponentially growing cells were diluted in the fresh isotonic NB containing 200 μg/mL DCS. The cells were incubated at 30°C without shaking. The phase contrast and DAPI staining nucleoid images were captured by several time points as indicated. Arrowheads indicate appearance of dead cells with phase pale effect. Scale bars represent 5 μm.

To confirm if RodA overproduction could work to stimulate L-form escape without the need for aPBPs, we cultured *Δ4* mutant cells carrying an ectopic *P_xyl_*-*rodA* construct in liquid nutrient broth (NB) with or without xylose, and then diluted those cells in isotonic NB containing DCS. In the absence of xylose, cells retained their cylindrical shape for at least 60 min but also began to die, as indicated by both phase pale appearance (lysis, arrowheads) and extreme nucleoid condensation (Figure 5, −Xylose). This is consistent with no appearance of L-form colonies of *Δ4* mutant cells on L-form selective plates (Figures 1C, 4B). When RodA was overproduced, most cells again retained their cylindrical shape but in this case, neither lysis nor nucleoid condensation occurred, at least during the first hour (Figure 5, +Xylose/60 min). However, after further incubation (> 120 min), L-forms, began to emerge from the still cylindrical parental cells (Figure 5 i), consistent with the L-form growth on plates shown in Figure 4B. Figure 5 ii shows an example of a cell in which the nucleoid appears to have been passing out of the parental shell in association with L-form emergence. These results suggest that two distinct pathways of L-form emergence exist, depending on whether PG synthesis is being supported by the aPBP or RodA PG synthases. Presumably, in both cases the crucial event could be the formation of cell wall lesions large enough to accommodate the exit of an L-form containing an intact nucleoid. This also suggests that RodA overproduction can enable the elongation PG system to function at the reduced PG precursor availability exerted by DCS treatment, and that this can facilitate the autolytic activity needed for L-form escape.

### LytE is the major autolysin acting with the Rod system

**Figure 6:**
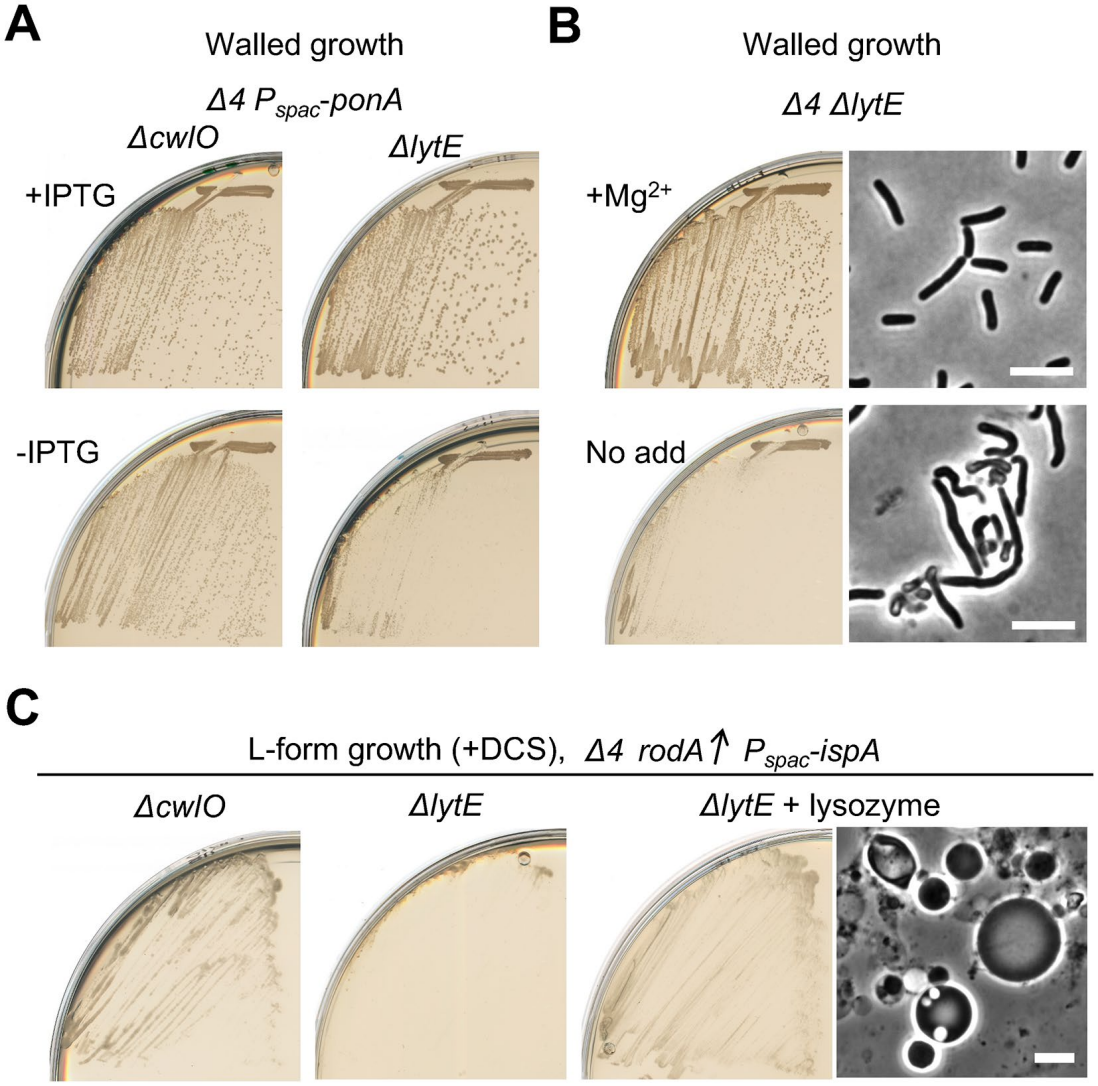
Crucial role for LytE during L-form escape by the Rod pathway **(A)** Growth of strains YK2562 (*Δ4 ΔcwlO P_spac_-ponA*) and YK2560 (*Δ4 ΔlytE P_spac_-ponA*) on NA plates with or without 0.5 mM IPTG (for *ponA* expression) at 30°C. **(B)** Growth and cell morphologies of strain YK2564 (*Δ4 ΔlytE*) on NA plates with or without added 10 mM Mg^2+^ at 30°C. Phase contrast micrographs were taken from colonies on the plates. Scale bars represent 5 μm. **(C)** L-form growth of strains YK2527 (*ΔcwlO Δ4 amyE*::*P_xyl_*-*rodA P_spac_*-*ispA*) and YK2526 (*ΔlytE Δ4 amyE*::*P_xyl_*-*rodA P_spac_*-*ispA*) on isotonic NA plates containing 1% xylose and 200 μg/mL DCS at 30°C for 2–3 days. YK256 was also cultured in the presence of 100 μg/mL lysoyme as indicated. Phase contrast micrograph was taken from the plate. Scale bars represent 5 μm.

Given that L-form escape is dependent on the aPBPs PG synthases, as well as the autolytic enzymes, as described above (Figures 1C, 2), autolytic enzymes could be also crucial in L-form escape by the Rod system in the absence of aPBPs. Since LytE and CwlO were obvious candidates, we introduced *ΔlytE* and *ΔcwlO* mutations separately into a *Δ4* strain that carried an IPTG inducible copy of the major aPBP gene, *ponA*. Figure 6A shows that the single deletion of *cwlO* had little if any effect on cell growth, with or without expression of *ponA* (left panels). In sharp contrast, however, repression of *ponA* in the strain with a deletion of *lytE* severely impaired growth (Figure 6A, right panels), suggesting that LytE has a more important role than CwlO during growth in cells lacking aPBPs. This would be consistent with the suggestion that LytE function is associated with the Rod system (Carballido-Lopez et al., 2006; Patel et al., 2020).

We then tested whether L-form escape also required the LytE. (Note that, during the course of experiments, we found that the growth deficiency of a *ΔlytE Δ4* mutant was suppressed in the presence of extra Mg^2+^ (Figure 6B) as seen for various elongation mutants (Formstone and Errington, 2005; Leaver and Errington, 2005; Schirner and Errington, 2009; Kawai et al., 2011), and taking advantage of this we generated a *ΔlytE Δ4* strain carrying an ectopic copy of P_xyl_-*rodA*) Figure 6C shows that the deletion of *lytE* largely prevented the emergence of L-form colonies. In contrast, no strong effect of *ΔcwlO* on L-form escape was observed. The escape defect in the *ΔlytE Δ4* mutant was rescued by the addition of an exogenous PG hydrolase, lysozyme, leading to the emergence of L-form colonies (Figure 6C, right), confirming a crucial role for LytE activity for the escape via the Rod pathway. Thus, just as for growth of walled cells, activity of the Rod system in supporting L-form escape was largely dependent on LytE, rather than the CwlO autolysin when the aPBP system did not operate.

## Discussion

Previous work from this lab had shown that one or other of LytE and CwlO are required for the walled to L-form switch that occurs when PG precursors are depleted (Kawai et al., 2018). In this situation, aPBPs are also required for the L-form switch, whereas the Rod system is not. We have now extended these findings by showing that depletion of PG precursors *via* antibiotic treatment or genetic repression of *glmM* also promotes L-form escape *via* the aPBP-dependent bulging process (Figure 3B) and that mutations affecting various other central components of the Rod or divisome complexes (FtsW, FtsZ, MreBCD, Mbl and MreBH) are not required for escape (Figure 2). Given that the L-form escape process requires both aPBPs and autolytic enzymes, we assume that bulging involves both PG synthetic and hydrolytic activities. Recent work with *E. coli* suggested that aPBP activity works to enhance the recovery of cells following cell wall inhibition (Vigouroux et al., 2020). In *B. subtilis*, cell width seems to be determined by the balanced activities of the Rod and aPBP systems (Dion et al., 2019). Increased Rod system activity associates with thinner cells and a more ordered PG matrix, but aPBP activity associates with increased width and less oriented material in the wall (Dion et al., 2019). The aPBPs may fill in the gaps in a foundational structure of oriented PG laid down in the cell cylinder by the Rod system to strengthen and expand its width (Rohs and Bernhardt, 2021). It has also been reported that assembly of the Rod elongation complex is dependent on the cellular abundance of the PG precursors in *B. subtilis* (Schirner et al., 2015). Based on our results we suggest an alternative or additional role for the aPBP system in supporting continued PG expansion when precursor availability is reduced. Nevertheless, the Rod elongation complex can clearly still work to support L-form escape in the absence of aPBP activity at low PG precursor levels, when RodA is overproduced (Figures 4, 5). Elucidating how the levels of PG precursors selectively influence the PG synthetic systems, thereby impacting on the mode of PG synthesis and L-form escape, is an important challenge for future.

As mentioned above, overproduction of RodA could rescue L-form generation in a *Δ4* mutant background during DCS treatment (Figure 4). Under these circumstances, bulging growth did not occur and the cells retained an approximately cylindrical shape (Figure 5). Nevertheless, L-form escape did occur, often from polar or approximately mid-cell locations. We previously reported that a strain with what turned out to be a complex mutant background, 168ED, also gave rise to L-forms, albeit less efficiently than in the experiments described here. In that case, L-form emergence also occurred without extensive cell bulging. The best understood mutation conferring this phenotype lay in the *sepF* gene (Domínguez-Cuevas et al., 2012). *sepF* mutations give rise to defective malformed division sites (Gündoğdu et al., 2011). Taken together with our new findings, we suggest that L-form escape requires the formation of a substantial cell wall lesion large enough to allow the passage of an L-form cell and its nuclear and cytoplasmic components, without serious damage to the chromosome or spillage of contents. It will be interesting in the future to explore the mechanisms involved in the successful exit of L-forms from their walled parent cells.

The cell wall-independent growth of L-forms allowed us to test whether either or both of the “essential” autolysins was required for L-form escape. As shown in Figure 6C, LytE was required but CwlO not. Thus, just as for growth of vegetative cells, it appears that LytE is partially specialised for functioning in the Rod pathway during L-form escape. Further work is needed to elucidate the molecular mechanisms coupling synthetic and autolytic activities during normal cell growth when these activities are balanced. Our results suggest that L-form escape will provide a useful experimental system for studying the coupling when lytic activity becomes dominant.

## Data availability statement

The original contributions presented in the study are included in the article/supplementary material, further inquiries can be directed to the corresponding author.

## Author contributions

YK designed concepts, performed experiments, and wrote the original draught of the manuscript. JE designed concepts, reviewed and edited the manuscript, and funding acquisition. All authors contributed to the article and approved the submitted version.

## Funding

This work was supported by a European Research Council Advanced award (670980), a Wellcome Investigator Award (209500) and an ARC Laureate Fellowship (FL210100071).

## Conflict of interest

The authors declare that this work carried out in the absence of any personal, commercial or financial relationships that could potentially be construed as a conflict of interest.

## Publisher’s note

All claims expressed in this article are solely those of the authors and do not necessarily represent those of their affiliated organizations, or those of the publisher, the editors and the reviewers. Any product that may be evaluated in this article, or claim that may be made by its manufacturer, is not guaranteed or endorsed by the publisher.

## Footnotes

^1^ https://imagej.net/Fiji
